# Transrectal versus transperineal prostate biopsy in detection of prostate cancer: a retrospective study based on 452 patients

**DOI:** 10.1186/s12894-023-01176-y

**Published:** 2023-01-28

**Authors:** Mengxin Lu, Yi Luo, Yongzhi Wang, Jingtian Yu, Hang Zheng, Zhonghua Yang

**Affiliations:** Department of Urology, Zhongnan Hospital of Wuhan University, 169 Donghu Road, Wuchang District, Wuhan, 430071 Hubei China

**Keywords:** Transrectal biopsy, Transperineal biopsy, Prostate cancer, Cancer detection rate, Positive rate of biopsy cores

## Abstract

**Background:**

Transrectal (TR) ultrasound guided prostate biopsy and transperineal (TP) ultrasound guided prostate biopsy are the two most commonly used methods to detect prostate cancer, the detection rate of the two biopsy approaches may differ in patients with different clinical characteristics. Here we aimed to compare the prostate cancer detection rate and positive rate of biopsy cores between TR and TP prostate biopsy in patients with different clinical characteristics.

**Methods:**

We retrospectively analyzed and compared the clinical data of 452 patients underwent TR or TP prostate biopsy in our hospital from June 2017 to September 2021. And patients were stratified according to several clinical characteristic (serum PSA level, prostate volume, PSA density, T stage and ISUP grade), cancer detection rate and positive rate of biopsy cores were compared in different stratified groups.

**Results:**

There was no significant difference in age, PSA level, prostate volume, and PSA density between the TR and TP groups. TR group had a higher overall cancer detection rate and positive rate of biopsy cores than TP group. Further subgroup analysis showed that TR group had a higher cancer detection rate in patients with prostate volumes 30–80 mL, and that the TR group had a higher positive rate of biopsy cores among the patients with T3–T4 stages, while TP group had a higher positive rates of biopsy cores among the patients with T1–T2 stages. There were no significant differences between the TR and TP groups for each subgroup when stratified by PSA level, PSA density and ISUP grade.

**Conclusions:**

TR approach may have advantage in patients with prostate volumes 30–80 mL and T3–T4 stages, while TP approach may have advantage in patients with T1–T2 stages.

## Background

Prostate cancer diagnosis rate is increasing year by year [1]. In 2021, prostate cancer is the first most common malignancy diagnosed in men worldwide [2]. When prostate cancer is suspected, prostate biopsy remains the standard method of diagnosis [3]. Transrectal (TR) ultrasound guided biopsy was first reported by Cooner et al. and further refined by Hodge et al*.* [4, 5]. Transperineal (TP) prostate biopsy offered an alternative approach, and after decades of being described, TP prostate biopsy becomes more and more widely adopted for its lower risk of infectious complications and higher diagnosis rate for anterior cancers [6, 7].

Several studies were performed to compare the detection rates and complications of TR and TP approaches. Firstly, TP biopsy significantly reduces infectious complications compared to TR biopsy [8–10], as well as improves the detection rate of anterior zone cancers [11, 12]. Moreover, there were no significant differences in both cancer detection rate and cancer core rate between TR and TP approaches for prostatic biopsy in most studies [13–16]. However, the detection rate of the two biopsy approaches may differ in patients of different ages, PSA values, and prostate volumes [17, 18].

12 cores biopsy was recommended to achieve the balance between the cancer detection rate and adverse events [19]. In our center, almost all prostate biopsies are performed by using 12 cores systematic biopsies plus 1 core in suspicious area. Transrectal prostate biopsy was the only choice until May 2020 in our hospital, and our center started performing free-hand TP ultrasound guided prostate biopsy in May 2020. In this study, the clinical data of 452 patients in our hospital who underwent prostate biopsy were retrospectively analyzed, to compare the detection rates between TP and TR approaches. In addition, by comparing detection rates of patients with different clinical characteristics, we aimed to find out the optimal biopsy approach in patients with different clinical characteristics.

## Material and methods

### Patient cohort

This study collected 452 patients underwent TR or TP prostate biopsy at the Department of Urology, Zhongnan Hospital of Wuhan University from June 2017 to September 2021, Patients with PSA ≥ 100 ng/mL were excluded because their prostate cancer detection rate was close to 100%. All methods used for analysis in this study were carried out in accordance with the approved regulations of the Medical Ethics Committee, Zhongnan Hospital of Wuhan University. The indications for prostate biopsy are based on ‘Chinese diagnosis and treatment of urological diseases Guide’, including patients with: (1) suspicious lesions detected by digital rectal examination (DRE); (2) suspicious lesions on imaging; (3) PSA > 10 ng/mL; (4) 10 ng/mL ≥ PSA > 4 ng/mL and abnormal free/total PSA ratio or PSA density.

### Biopsy procedure

Almost all prostate biopsies (both TR and TP approaches) in our hospital are performed by using 12 cores systematic biopsies plus 1 core in suspicious area. Biopsies were accomplished in a “fan-like” pattern [20], an 18-gauge disposable core biopsy instrument (Bard Healthcare Science, Shanghai, CO, LTD.) was used to obtain specimens. Each core was marked and packed site-specifically, and assigned a separate Gleason score. And prostate biopsy is mainly performed by Dr. Yi Luo and Dr. Zhonghua Yang, who are good at the both two biopsy methods. Transrectal prostate biopsy was the only choice until May 2020. Ever since our hospital performed free-hand TP ultrasound guided prostate biopsy in May 2020, TP biopsy is preferentially recommended for patients because of its lower infection rate according to the current European Association of Urology (EAU) guidelines. And transrectal prostate biopsy, with lower cost because of no requirement for anesthesia, was an alternative approach when the patient chose it.

**Fig. 1:**
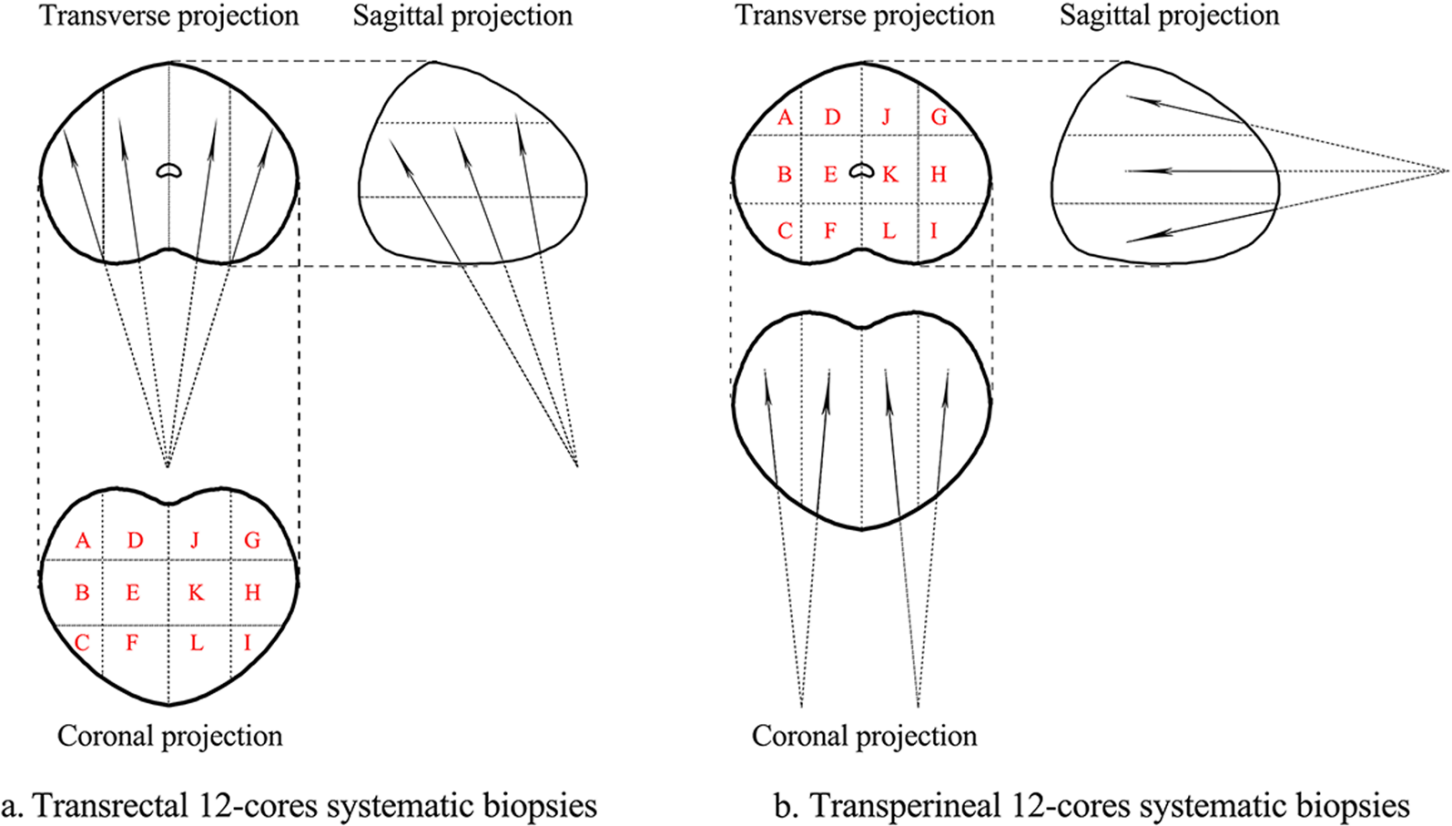
Transrectal and transperineal prostate biopsy model diagram: transverse, sagittal, and coronal projections of the TR (**a**) and TP (**b**) 12-cores systematic biopsies

In accordance with the same steps reported in the literature [21], the patient who underwent transrectal procedure was placed in the left lateral decubitus position. No anesthesia was administered. A transrectal ultrasound (TRUS) probe was used for needle placement and guidance. A standard biopsy procedure sampled parasagittal midline and lateral apical, medial, and basal regions bilaterally for a total of 12 cores as well as the additional cores from areas of suspicion (Fig. 1a). The patient who underwent transperineal procedure was positioned in lithotomy and general anesthesia was administered. Excessive hair was shaved and the perineum was prepared using 1% povidone-iodine. An indwelling Foley catheter was used to visualize the urethra on ultrasound images. The scrotum was elevated anteriorly using surgical incise drape to expose the perineum. A curvilinear ultrasound probe (transperineal ultrasound, TPUS) was pressed vertically against the lower part of the perineum and visualized the gland. The needle was inserted from the upper edge of the probe in plane with the ultrasound beam. The prostate was divided into left and right lobes. For each lobe, six cores were taken from anterior, middle and posterior zones of the lateral and medial area, additional cores were taken from areas of suspicion (Fig. 1b).

### Data collection

The following clinical data was acquired by retrospective review of medical records and compared between the two groups: age, serum PSA level (ng/mL), prostate volume (mL), prostate cancer diagnosis rate and positive rate of biopsy cores, the prostate volume was calculated using the formula: height × width × length × 0.52 (in cm). Furtherly, patients were stratified according to several clinical characteristic, including: serum PSA level, prostate volume, PSA density, T stage and ISUP (international society for urological pathology) grade, cancer detection rate and positive rate of biopsy cores were compared in different stratified groups.

### Statistical analyses

Continuous variables were described as averages, ranges and medians. Age was compared by independent t test, while PSA level, prostate volume and PSA density were compared by Mann–Whitney U test as they did not conform to normal distribution. The normal distribution of each dataset was checked by Kolmogorov–Smirnov test. Chi-square test was performed to compare the prostate cancer diagnosis rate and positive rate of biopsy cores among different groups. Cochran–Mantel–Haenszel (CMH) test was performed to compare the cancer detection rate after controlling for confounding variables according to PSA level and PSA density. The results were expressed as odds ratios (ORs) with 95% confidence intervals (CIs). SPSS 22.0 was used to perform all statistical analyses and *p* value < 0.05 was considered significant.

## Results

Out of the 452 patients underwent prostate biopsy (Table 1), 230 (50.9%) cases were diagnosed with prostate cancer, the overall positive rate of biopsy cores was 24.6% (1416 of 5759 cores). Average age was 70.1 years, average PSA level was 22.0 ng/mL, average prostate volume was 57.0 mL, and average PSA density was 0.51 ng/mL^2^. There was no significant difference in age, PSA level, prostate volume, and PSA density between two groups. The overall detection rate in TR group appeared to be higher than in TP group in our study (56.3% vs 44.4%, OR: 1.612, 95% CI: 1.111–2.340, *p* = 0.012). To minimize confounding factors of PSA level and PSA density in two groups, CMH test was performed, showing that the cancer detection rate in TR group was still higher than in TP group both when adjusting with PSA level (OR: 1.529, 95% CI: 1.036–2.255, *p* = 0.032), or when adjusting with PSA density (OR: 1.514, 95% CI: 1.030–2.226, *p* = 0.035). Similarly, the positive rate of biopsy cores was higher in the TR group than in the TP group (27.5% vs 21.0%, OR: 1.429, 95% CI: 1.264–1.616, *p* < 0.001).

**Table 1:** Clinical characteristics of patients underwent transrectal or transperineal prostate biopsy

Variables	All patients (n = 452)	Transrectal biopsy (n = 245)	Transperineal biopsy (n = 207)	*p* value
*Age at biopsy (years)*				
Average/Median	70.1 ± 8.4/70	70.0 ± 8.7/70	70.3 ± 8.1/70	0.769
Range	45–88	45–88	51–88	
*PSA (ng/mL)*				
Average/Median	22.0 ± 20.6/13.8	23.2 ± 21.8/14.1	20.6 ± 19.0/13.7	0.634
Range	0.3–99.2	0.3–99.2	0.8–97.6	
*Prostate volume (mL)*				
Average/Median	57.0 ± 36.0/49	54.4 ± 31.6/46	60.0 ± 40.3/52	0.151
Range	10–334	11–204	10–334	
*PSA density (ng/mL*^*2*^*)*				
Average/Median	0.51 ± 0.59/0.27	0.55 ± 0.64/0.29	0.47 ± 0.51/0.26	0.181
Range	0.01–3.63	0.01–3.63	0.02–2.40	
Prostate cancer diagnosis rate (n, %)	230/452 (50.9%)	138/245 (56.3%)	92/207 (44.4%)	0.012
Positive rate of biopsy cores (n, %)	1416/5759 (24.6%)	876/3185 (27.5%)	540/2574 (21.0%)	< 0.001

Furthermore, to compare the prostate cancer detection rate of two biopsy approaches in patients with different clinical characteristics, subgroup analysis was performed according to serum PSA level, prostate volume and PSA density. As shown in Table 2, In subgroup analysis stratified by PSA level, there were no significant differences in cancer detection rate between the TR and TP groups for each subgroup. When stratified by prostate volume, the TR group have a higher cancer detection rate than the TP group among the patients with 30–80 mL prostate volume (56.3% vs 40.5%, OR: 1.892, 95% CI: 1.165–3.073, *p* = 0.010), as well as in the subgroup with > 80 mL prostate volume (36.6% vs 16.3%, OR: 2.967, 95% CI: 1.060–8.305, *p* = 0.034). However, there was no significant difference between two biopsy approaches in cancer detection rate among the patients with ≤ 30 mL prostate volume. Similarly, cancer detection rates did not significantly differ between TR and TP group according to different PSA density. To minimize confounding factors of PSA level in subgroup analysis with different prostate volume, CMH test was performed, showing that TR group still have a higher cancer detection rate than the TP group with 30–80 mL prostate volume (OR: 1.838, 95% CI: 1.086–3.112, *p* = 0.023), while there was no significant difference in cancer detection rate in the subgroup with > 80 mL (*p* = 0.087).

**Table 2:** Comparison of prostate cancer detection rate according to PSA level, prostate volume and PSA density between transrectal and transperineal prostate biopsy

Variables	All patients (n, %)	Transrectal biopsy (n, %)	Transperineal biopsy (n, %)	*p* value
*PSA (ng/mL)*				
≤ 10	63/152 (41.4)	40/87 (46.0)	23/65 (35.4)	0.190
10–20	56/140 (40.0)	29/63 (46.0)	27/77 (35.1)	0.188
20–100	111/160 (69.4)	69/95 (72.6)	42/65 (64.6)	0.280
*Prostate volume (mL)*				
≤ 30	74/96 (77.1)	38/53 (71.7)	36/43 (83.7)	0.163
30–80	134/272 (49.3)	85/151 (56.3)	49/121 (40.5)	0.010
> 80	22/84 (26.2)	15/41 (36.6)	7/43 (16.3)	0.034
*PSA density (ng/mL*^*2*^*)*				
≤ 0.15	23/92 (25.0)	14/42 (33.3)	9/50 (18.0)	0.091
> 0.15	207/360 (57.5)	124/203(61.1)	83/157 (52.8)	0.118

Among 230 patients diagnosed with prostate cancer by biopsy, T stages were assessable in 175 patients (104/138 in TR group, 71/92 in TP group). We further analyzed the difference in the positive rates of biopsy cores between the two biopsy approaches in patients with different T stages. As shown in Table 3, the TR group have a higher positive rates of biopsy cores among the patients with T3 and T4 stages (72.4% vs 62.5%, OR: 1.573, 95% CI: 1.213–2.038, *p* = 0.001), while TP group have a higher rate among the patients with T1 and T2 stages (30.6% vs 36.9%, OR: 0.754, 95% CI: 0.585–0.972, *p* = 0.029). Additionally, except for 1 patient in TR group diagnosed with neuroendocrine carcinoma, ISUP (international society for urological pathology) grades were obtained according to the highest pathology score core for each individual patient. When stratified by ISUP grade, there were no significant differences in positive rates of biopsy cores between the TR and TP groups for each subgroup.

**Table 3:** Comparison of positive rate of biopsy cores according to T stage and ISUP grade between transrectal and transperineal prostate biopsy

Variables	All patients (n, %)	Transrectal biopsy (n, %)	Transperineal biopsy (n, %)	*p* value
*T stage*				
T1 + T2	381/1160 (32.8)	231/754 (30.6)	150/406 (36.9)	0.029
T3 + T4	725/1065 (68.1)	433/598 (72.4)	292/467 (62.5)	0.001
*ISUP grade*				
1	79/542 (14.6)	49/351 (14.0)	30/191 (15.7)	0.582
2–3	369/853 (42.3)	192/455 (42.2)	177/398 (44.5)	0.504
4–5	955/1520 (62.8)	622/975 (63.8)	333/545 (61.1)	0.297

## Discussion

Since TR ultrasound guided biopsy was introduced by Hodge et al. [5], it has gradually become the main approach for the diagnosis of prostate cancer worldwide. Despite the routine use of antimicrobial prophylaxis, approximately 3–5% of TR approach biopsy patients are readmitted for infection [22, 23]. TP ultrasound guided biopsy has been recommended as a safer alternative for its lower infection rate [9, 10]. In our study, 4.1% (10/245) of patients in the TR group developed fever (> 38.0 °C) following the biopsy procedure, while 1.0% (2/207) in the TP group developed fever. Statistically, patients had a higher infection rate in TR group than in TP group (OR: 4.362, 95% CI: 0.945–20.137, *p* = 0.040). Because of the lower infection rate of TP biopsy according to the current EAU guidelines, TP biopsy is preferentially recommended for patients ever since it was performed in our hospital in May 2020. And for this reason, more than 90% of patients preferred to choose TP biopsy when available, while a few patients chose TR biopsy, which is less expensive with no need for anesthesia. Over the past decades, with the improvement of multiparametric MRI (magnetic resonance imaging) diagnosis, MRI has become more and more sensitive for the detection of prostate cancer [24]. MR/ultrasound fusion-guided biopsy (combined targeted biopsy and systematic biopsy) appeared to detect more cases of prostate cancers than ultrasound-guided biopsy, especially clinically significant cancer [25, 26]. However, because of the high cost of MR/ultrasound fusion-guided biopsy, TRUS-guided systematic biopsy is still the primary method of prostate biopsy for its easy promotion.

Several previous studies were performed to compare the prostate cancer detection rates of TR and TP approaches, indicating no significant difference in overall cancer detection rates between the two approaches [13, 14, 27–29]. Jiang et al. [17] reported that the detection rates of TR approach were higher than TP approach for patients with PSA level 20.1–100.0 ng/mL (80.8% vs 69.1%, *p* = 0.040). In our study, the overall detection rate in TR group appeared to be higher than in TP group (56.3% vs 44.4%, OR: 1.612, *p* = 0.012). We speculate that this difference may be due to the average PSA level (22.0 ± 20.6 ng/mL) in our study were significantly higher than in the most previously reported studies[13–17, 27–29]. In addition, because curvilinear ultrasound probe is easier to obtain, with no need for bowel preparation, and more easily popularized in primary level hospital, TP prostate biopsy in our study was carried out in a free-hand fashion, and under transperineal ultrasound probe guidance, which may be another reason for the different overall detection rate between TR and TP group in our study. However, when stratified by PSA level, there were no significant differences in cancer detection rate between the TR and TP groups for patients with 20–100 ng/mL PSA level (72.6 vs 64.6%, *p* = 0.280) in our study, which may be caused by the small sample size in the subgroup.

In China, the proportion of newly diagnosed prostate cancer patients with high-risk and advanced-stage prostate cancer is higher than in Europe and the United States, which is more prevalent in poorer areas [30]. Among 410 patients diagnosed with prostate cancer in our hospital (180 patients whose prostate volume was not available, or with PSA ≥ 100 ng/mL were excluded from the study), patients with PSA levels > 20 ng/mL accounted for 68.3% (280/410), patients with ISUP grade 4–5 accounted for 66.3% (272/410), and patients with T3 and T4 stages accounted for 67.2% (197/293). Consistent with the higher diagnostic rate of TR approach in patients with PSA > 20 ng/mL reported in the literature [17], the TR group had a higher positive rate of biopsy cores among the patients with T3 and T4 stages (72.4% vs 62.5%, OR: 1.573, *p* = 0.001) in our study, which suggested that TR approach may have advantage in patients with a probable diagnosis of high-risk prostate cancer (PSA levels > 20 ng/mL, cT3–T4 stage tumor on imaging or digital rectal examination). In contrast, TP group seems to have a higher positive rates of biopsy cores among the patients with T1 and T2 stages (30.6% vs 36.9%, OR: 0.754, p = 0.029), and the literature also reported the advantage of the TP approach in patients with PSA levels of 4.01–10.00 ng/mL [6, 27], indicating that TP approach may have advantage in patients with low- and intermediate-risk prostate cancer. The higher positive rates of biopsy cores of TP biopsy in T1–T2 stages may result from the advantage of TP biopsy for detecting anterior zone cancers. While in TR biopsy, because its needles entered the prostate gland from the back of the gland, more of the biopsy cores pass through the large prostate cancers in peripheral area, which may be the reason for its advantage in the patients with T3 and T4 stages.

In our results, TR group had a higher cancer detection rate than TP group in patients with prostate volumes 30–80 mL (OR: 1.838, 95% CI: 1.086–3.112, *p* = 0.023). As we known, the enlargement of prostate volume was mainly due to the hyperplasia of transitional zone, which will cause the peripheral zone (an area with a high incidence of prostate cancer) to be compressed to the back of the gland. When TR biopsy was applied in the hyperplastic prostate, most cores will pass through the peripheral zone because its needles entered the prostate gland from the back of the gland. While the ventral needles (A, D, J, G in Fig. 1) of TP biopsy will miss the peripheral zone in the hyperplastic prostate, because its needles entered the prostate gland from the apex of prostate. This may be the reason for the higher cancer detection rate of TR group in patients with prostate volumes 30–80 mL. Koparal et al*.* reported that TP biopsy had a higher cancer detection rate than TR group in patients with prostate volumes > 100 mL (average PSA level 6.6 ng/mL) [18]. Moreover, when the prostate volume increases to more than 80 mL, there was no significant difference in cancer detection rate between two biopsy approaches in our study. We speculate that the limitation and the more intense pain of the transrectal biopsy in patients with large prostate volumes may be the reason for this result [31].

Our study had some limitations. Firstly, this is a single-center retrospective study with a low level of evidence. Additionally, partial information was missing for some patients in this study, such as T-stage. More research is needed to further verify these opinions.

## Conclusions

Based on 452 patients underwent prostate biopsy in our hospital, TR group had a higher overall cancer detection rate and positive rate of biopsy cores than TP group. Further subgroup analysis showed that TR group had a higher cancer detection rate in patients with prostate volumes 30–80 mL, and that the TR group had a higher positive rate of biopsy cores among the patients with T3–T4 stages, while TP group had a higher positive rates of biopsy cores among the patients with T1–T2 stages. There were no significant differences between the TR and TP groups for each subgroup when stratified by PSA level, PSA density and ISUP grade.

## Data Availability

The datasets used or analysed during the current study are available from the corresponding author on reasonable request.

## Abbreviations

TR: Transrectal
TP: Transperineal
PSA: Prostate-specific antigen
DRE: Digital rectal examination
TRUS: Transrectal ultrasound
TPUS: Transperineal ultrasound
ISUP: International society for urological pathology
CMH: Cochran–Mantel–Haenszel
OR: Odds ratio
CI: Confidence interval
MRI: Magnetic resonance imaging
